# Patient-derived SARS-CoV-2 mutations impact viral replication dynamics and infectivity in vitro and with clinical implications in vivo

**DOI:** 10.1038/s41421-020-00226-1

**Published:** 2020-10-29

**Authors:** Hangping Yao, Xiangyun Lu, Qiong Chen, Kaijin Xu, Yu Chen, Minghui Cheng, Keda Chen, Linfang Cheng, Tianhao Weng, Danrong Shi, Fumin Liu, Zhigang Wu, Mingjie Xie, Haibo Wu, Changzhong Jin, Min Zheng, Nanping Wu, Chao Jiang, Lanjuan Li

**Affiliations:** 1 State Key Laboratory for Diagnosis and Treatment of Infectious Diseases, National Clinical Research Center for Infectious Diseases, First Affiliated Hospital, Zhejiang University School of Medicine, Hangzhou, Zhejiang 310009 China; 2 Life Sciences Institute, Zhejiang University, Hangzhou, Zhejiang 310012 China; 3 Shulan International Medical College, Zhejiang Shuren University, Hangzhou, Zhejiang 310015 China; 4 Zhejiang Provincial Key Laboratory of Pancreatic Disease, First Affiliated Hospital, Zhejiang University School of Medicine, Hangzhou, Zhejiang 310009 China

**Keywords:** Molecular biology, Cell biology

## Abstract

The severe acute respiratory syndrome coronavirus 2 (SARS-CoV-2) has spread globally with more than 33 million patients diagnosed, taking more than a million lives. Abundant mutations were observed but the functional consequences of these mutations are largely unknown. We report the mutation spectrum, replication dynamics, and infectivity of 11 patient-derived viral isolates in diverse cell lines, including the human lung cancer cell line Calu-3. We observed 46 mutations, including 9 different mutations in the spike gene. Importantly, these viral isolates show significant and consistent variations in replication dynamics and infectivity in tested cell lines, up to a 1500-fold difference in viral titers at 24 h after infecting Calu-3 cells. Moreover, we show that the variations in viral titers among viral isolates are positively correlated with blood clotting function but inversely correlated with the amount of red blood cell and hemoglobin in patients. Therefore, we provide direct evidence that naturally occurring mutations in SARS-CoV-2 can substantially change its replication dynamics and infectivity in diverse human cell lines, with clinical implications in vivo.

## Introduction

Severe acute respiratory syndrome coronavirus 2 (SARS-CoV-2) has caused a global pandemic. As of September 29, 2020, SARS-CoV-2 has infected more than 33 million people around the world with a death toll growing to more than a million. The numbers are still increasing rapidly. More than half of patients with SARS-CoV-2 were asymptomatic^1^, who can still transmit the Coronavirus Disease-2019 (COVID-19) disease^2,3^, making it much more challenging to prevent. There is clearly an urgent need to develop effective vaccines or antibody-based therapeutics against SARS-Cov-2^4,5^. According to the report from the World Health Organization, there are currently 40 candidate vaccines in clinical evaluation and 151 candidate vaccines in preclinical development (https://www.who.int/publications/m/item/draft-landscape-of-covid-19-candidate-vaccines, accessed September 29th, 2020).

The first reported successful isolation of the SARS-CoV-2 virus is from COVID-19 cases in Wuhan. SARS-CoV-2 is the seventh member of enveloped RNA coronavirus (Sarbecovirus subgenus) known to infect humans. The transmembrane Spike (S) glycoprotein mediates viral entry into host cells through homotrimers protruding from the viral surface. The S protein includes two domains: S1 for binding to the host cell receptor, and S2 for a fusion of the viral and cellular membranes^6^. Both SARS-CoV-2 and SARS-CoV use the angiotensin-converting enzyme 2 (ACE2) to enter target cells^7^. ACE2 is expressed in a variety of human lung cells^8^, cholangiocytes^9^, kidney cells^10^, etc.

The receptor-binding domain (RBD) in the S protein is the most variable genomic part in the betacoronavirus group^11,12^. Some sites around the RBD in the S protein appear subject to purifying selection among bat, pangolin, and human coronaviruses^13,14^, whereas other sites in the S protein have undergone positive selection, providing insights into common evolutionary mechanisms that could lead to new emerging human coronaviruses. SARS-CoV-2 mutates at a steady pace, roughly 23.6 mutations/year (GISAID). Despite the abundant variability of SARS-CoV-2^15,16^, one key question remains as to whether these mutations have any functional impact on the replication dynamics and infectivity of SARS-CoV-2. Researchers found that SARS-CoV-2 with natural elongation variant ORF3b may exacerbate COVID-19 symptoms^17^. In addition, recent studies report that the Europe-prevalent D614G mutation found in the S protein may lead to changes in the binding efficiency of the S protein using pseudo viral particles^16,18^. Detailed monitoring and studying of these naturally occurring mutations is crucial to our understanding of the virus, and can potentially inform drug and vaccine development strategies targeting specific mutable epitopes of SARS-CoV-2.

**Fig. 1:**
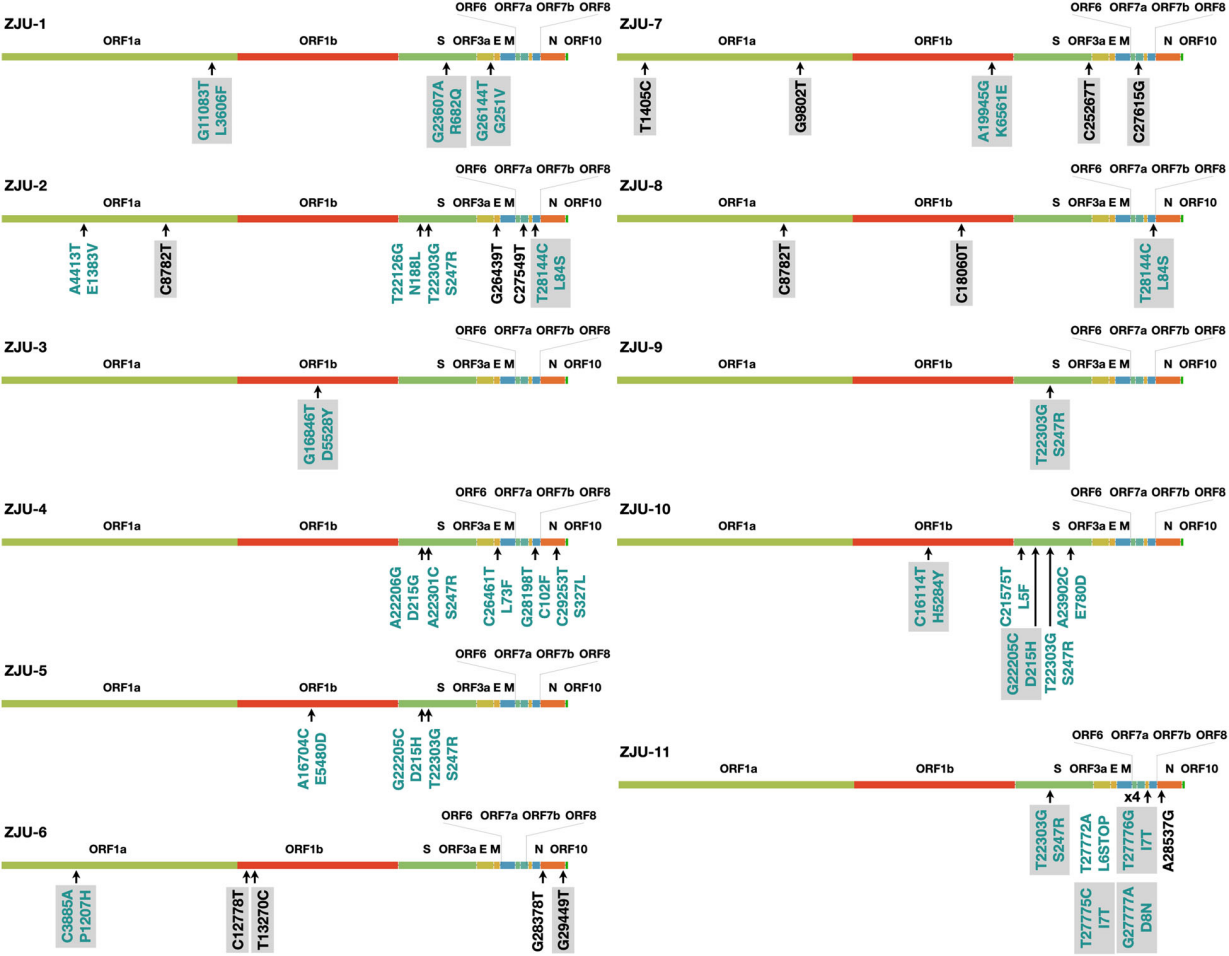
A summary of the mutations identified in 11 viral isolates. Each ORF of the viral genome was denoted based on the annotations of NC_045512.2 as provided by NCBI. Shades indicate consensus mutations (mutation frequency ≥ 50%). Teal text indicates nucleotide mutations that lead to amino acid changes (missense mutations) in respective gene products. Black text indicates synonymous mutations. Note that ZJU-11 has four mutations in the *ORF7b* gene. The lengths of genes were drawn proportionally.

To address this, we characterized 11 SARS-CoV-2 viral isolates from patients admitted to Zhejiang University-affiliated hospitals in Hangzhou, China, situated 757 kilometers to the east of Wuhan (see Materials and methods section). Ultra-deep sequencing of the 11 viral isolates on the Novaseq 6000 platform identified 1–7 mutations within each patient in the coding sequences among the viral isolates (Fig. 1). Extensive mixed viral populations (representing quasi-species) were also observed. We infected African green monkey kidney-derived Vero cell line, human lung cancer cell line Calu-3, and human liver cancer cell line Huh-7 with 11 patient-derived viral isolates and quantitatively assessed their viral titer and infectivity up to 72 h post-infection (P.I.). Our results showed that the identified mutations can have a direct impact on the viral titers and infectivity, with up to more than a 1500-fold difference between the extremities in the Calu-3 cells. Furthermore, we showed that the variations in viral titers among viral isolates are positively correlated with blood clotting function but inversely correlated with the amount of red blood cell and hemoglobin in patients. These results suggest that the observed mutations in our study, and possibly in the viral isolates collected around the world, could significantly impact the replication dynamics and infectivity of SARS-CoV-2, potentially contributing to the severity of the disease in patients.

## Results

### Summary of the epidemiological history of the patients

The samples of the 11 patients involved in this study were collected during the early phase of the COVID-19 outbreak in China, dates ranging from 1/22/2020 to 2/4/2020 (Supplementary Table S1). 10 of the 11 patients had clear connections with Wuhan city, where the first cases of COVID-19 in China were reported. 5 of the 11 people either worked in or traveled to Wuhan before they were diagnosed, and another 5 had close contact with people who lived in Wuhan, and the remaining person had contact with people who were COVID-19-positive. Notably, patients ZJU-4, -5, -9 attended the same business conference where a few colleagues from Wuhan were present. These patients, therefore, constitute 1st and 2nd generations of the viral victims based on their epidemiological history. The ages of the 11 patients were from 4 months to 71 years old. All except one of the patients had moderate or worse symptoms. Three patients had comorbidities and one patient needed ICU treatment. Luckily, all patients have recovered as of the time of writing this article.

### Ultra-deep sequencing reveals diverse mutations in the patient-derived viral isolates

To assess the mutational spectrum of these 11 viral isolates, ultra-deep sequencing of the isolated viral genomic RNA was performed on the Illumina Novaseq 6000 platform, generating on average 245 million post-cleaning reads/67.16 Gb per sample (Supplementary Table S2; average coverage exceeding 2,000,000×). In cases where the viral populations were not homogenous, the ultra-depth could help us to characterize low-frequency mutations.

**Fig. 2:**
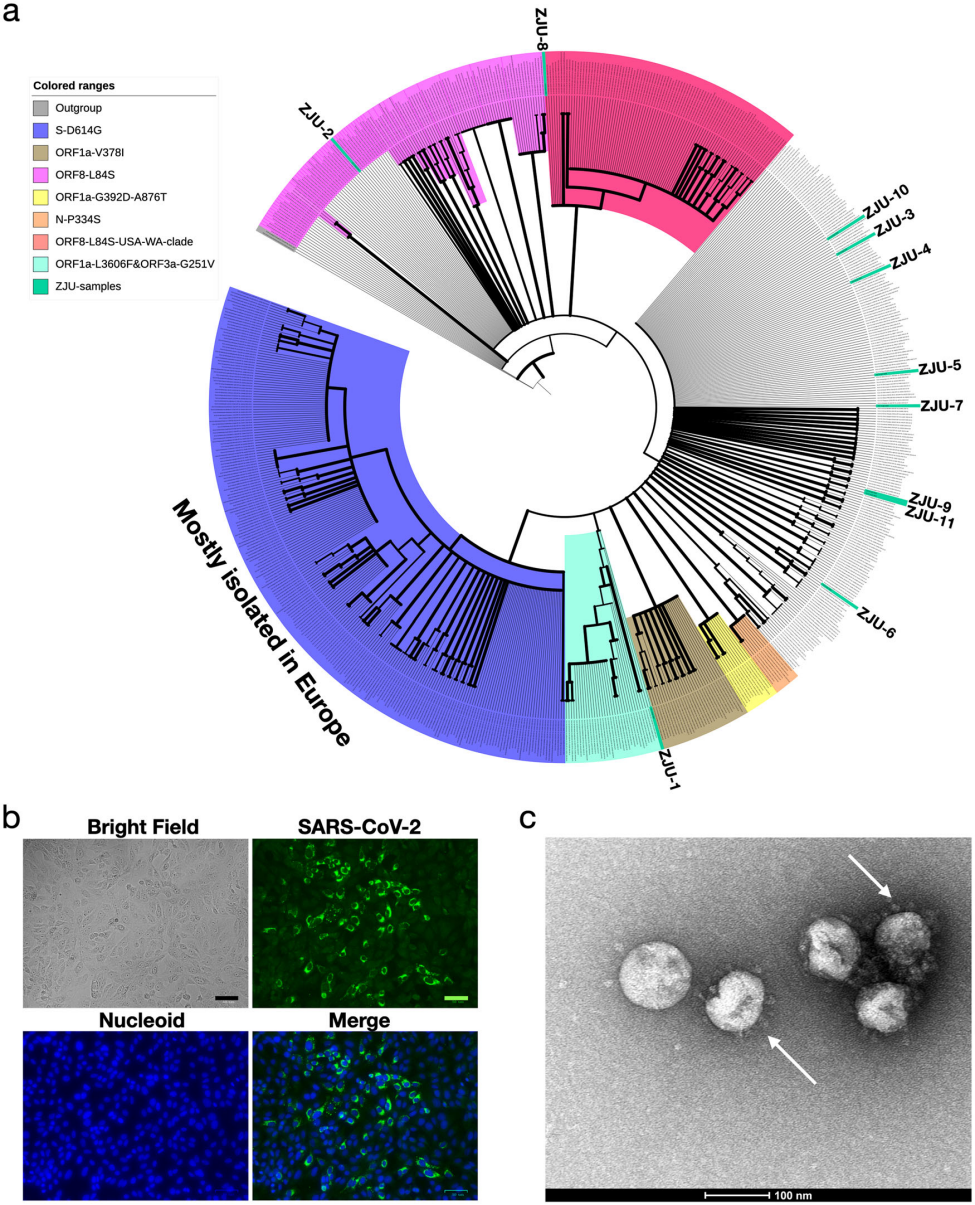
Characterizations of the patient-derived SARS-CoV-2 isolates. **a** Phylogenetic analysis of the 11 viral isolates in the context of 725 SARS-CoV-2 sequences downloaded from GISAID. Major and minor clusters were color-coded and denoted in the “colored ranges” inset box. All ZJU samples were color-coded as green. The width of a branch indicates the bootstrap supporting level. **b** Fluorescent labeling of the viral S protein indicates that isolated SARS-CoV-2 viral particles (green) assemble in the periphery of the Vero cells (DNA is stained as blue). Scale bars, 50 µm. **c** A representative TEM picture of the isolated SARS-CoV-2 viral particles; arrows indicate the iconic “crown” consisting of S proteins (spike). Scale bar, 100 nm.

In total, 46 mutations were identified, including 21 non-consensus mutations (frequency ≤ 50%) and 2 mutations in regulatory sequences (Figs. 1, 2a and Supplementary Figs. S1, S2, Table S3). Specifically, G11083T and G26144T were found in ZJU-1, and both of these mutations are shared by a large group of viral isolates^19^. C8782T and T28144C were found in ZJU-2 and ZJU-8, and these two are shared by another large group of viral isolates originally found in North America^19^. Interestingly, T22303G was found in five viral isolates (ZJU-2, -5, -9, -10, and -11), among whom ZJU-5 and ZJU-9 were exposed to SARS-CoV-2 at the same business conference (Supplementary Table S1). Previously, only one viral isolate identified in Australia had the T22303G mutation^20^. Strikingly, the viral isolate from patient ZJU-4, who attended the same conference as ZJU-5 and ZJU-9, had a novel mutation, A22301C, which causes the same missense mutation at the protein level (S247R in the S protein) as T22303G. Finally, the ZJU-11 isolate had four mutations in the *ORF7b* gene, three of which were consecutive, introducing two mutations at the protein level. Di-nucleotide and trinucleotide mutations were rarer than SNV, but not exponentially so, similar to results from previous mutational accumulation studies in prokaryotes^21^.

It is important to note that while the sequence data deposited in GISAID are very helpful in tracking inter-personal variations of the virus, we still do not know much about intra-personal viral evolutionary dynamics. For example, mutations at two separate genomic sites in ZJU-4 and ZJU-10 had very similar allele frequencies (Supplementary Table S3), indicating that these two sites are probably linked, representing a viral haplotype within the viral populations. 21 of the identified 46 mutations would have been ignored if only using consensus sequences for analyses. Taken together, despite only 11 patient-derived isolates being analyzed in this study, we observed abundant mutational diversity, including mutations shared by different major clusters of viruses now circulating globally. This diverse mutational spectrum is consistent with their relatively early sampling time and relative proximity to Wuhan city, where the first cases of COVID-19 in China were reported. Unfortunately, the full mutational diversity of the virus in Wuhan city in the early days is still unknown, due to limited sampling^22,23^.

### Phylogenetic analysis of the patient-derived viral isolates reveals their diverse evolutionary history

To understand the phylogenetic context of the 11 viral isolates with respect to the corpus of available SARS-CoV-2 sequencing data during the early phase of the pandemic, as the later global exchange would mask the earlier geo-based clustering patterns, we acquired 725 high-quality and high-coverage SARS-CoV-2 genomes from the GISAID database (downloaded on 3/21/2020), including the Yunnan RaTG13 viral strain and the Guangdong pangolin viral strain as the outgroup. The resulting phylogenetic tree was largely consistent with the phylogenetic analysis being updated on GISAID (see Materials and methods section, Fig. 2a, and Supplementary Fig. S3).

Specifically, we note the following three clusters: (1) three nucleotide mutations C241T (silent), C14408T (silent), and A23403G (D614G in S) were shared by a group of 231 viral isolates (Fig. 2a; S-D614G cluster), most of which were isolated in Europe; (2) two nucleotide mutations C8782T (silent) and T28144C (L84S in ORF8) were shared by a group of 208 viral isolates (Fig. 2a; ORF8-L84S cluster), which is not monophyletic in our analysis (Fig. 2a and Supplementary Fig. S3). However, a distinct monophyletic subclade of 92 viral isolates within the ORF8-L84S cluster can be observed, mainly composed of viral sequences isolated from Seattle, USA (Fig. 2a; ORF8-L84S-USA-WA-clade); (3) two nucleotide mutations, G11083T (L3606F in ORF1a) and G26144T (G251V in ORF3a) were shared by a group of 34 viral isolates, most of which were from the Netherlands and England. Several smaller monophyletic clusters were also observed (Fig. 2a).

The 11 viral isolates from this study were dispersed across the entire phylogenetic space. ZJU-1 clusters with the ORF1a-L3606F & ORF3a-G251V groups (Fig. 2a). ZJU-2 and ZJU-8, on the other hand, cluster with the ORF8-L84S cluster (Figs. 1 and 2a). ZJU-9 and ZJU-11 cluster with an Australian isolate because of the T22303G mutation. The rest of the group do not cluster with any known major groups, reflecting the extensive diversity within our 11 samples. Taken together, the mutations identified in these isolates were phylogenetically diverse, consistent with their early sampling dates.

### Patient-derived SARS-CoV-2 isolates show significant variations in replication dynamics in Vero cells

The human-to-human infectious process is a series of repeated naturally occurring bottlenecking events, in which the seeding viral population can be as small as hundreds of viral copies^24^. Therefore, a significant portion of the genetic diversity and population-specific fixations could be due to this process, where selection plays a small role^25^. We conducted Tajima’s test of neutrality using the constructed alignment of viral sequences, and Taijima’s D is −2.8874 with a nucleotide diversity (π) of 0.000641 (*P* < 0.05 according to simulations performed^26^), indicating that the SARS-CoV-2 genome has an excess of low-frequency alleles due to recent population expansions, consistent with the repeated bottlenecking events during viral infections. However, certain mutations do provide selection advantages or disadvantages under specific circumstances, as shown by the discovery that adaptive mutations are highly enriched in the interface between the S protein and the human ACE2 receptor^27^.

**Fig. 3:**
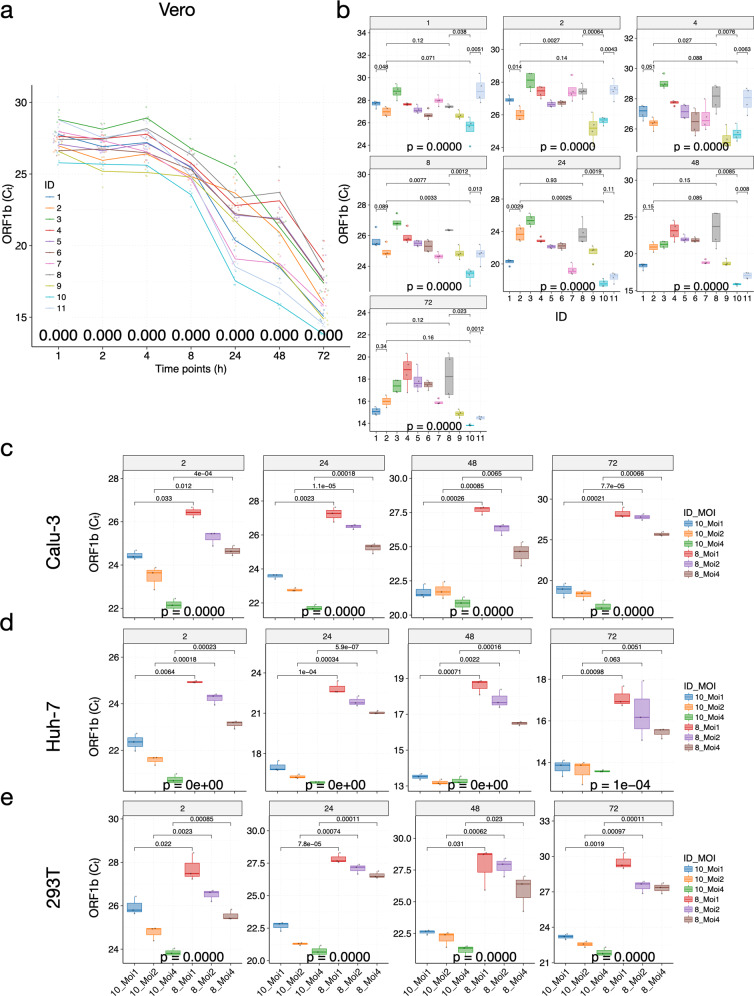
Patient-derived mutations in SARS-CoV-2 directly impact the viral replication dynamics in Vero cells. **a** Time-series plots of the *C*_t_ values (corresponding to the multiplicative inverse of the viral titer) of the SARS-CoV-2 *ORF1b* gene over the course of infection. **b** Significant variations in viral titers were observed at each timepoint among the 11 viral isolates. **c**–**e** Variations in viral titers when infected by ZJU-8 and ZJU-10 using different MOIs in human cancer cell lines Calu-3 (**c**), Huh-7 (**d**), and 293 T (**e**). Each viral isolate was color-coded accordingly. At each timepoint, a *P*-value was calculated using the ANOVA method to compare the means of *C*_t_ values of different viral isolates. Pairwise *P*-values were calculated using the *t*-test and adjusted *P*-values are shown.

To examine the impact of the different mutations in patient-derived SARS-CoV-2 isolates, we first confirmed whether viral isolates could successfully bind to Vero cells (Fig. 2b), and visually identified the viral particles with the iconic “crown” formed by S proteins (Fig. 2c). We then investigated the viral replication dynamics by infecting Vero cells with all 11 patient-derived viral isolates and harvested the cells (in quadruplicates) at 1, 2, 4, 8, 24, 48, and up to 72 h P.I. (see Materials and methods section). For all infection experiments, respective viral stocks were adjusted to the same multiplicity of infection (MOI = 0.5) based on the TCID_50_ of their clinical samples; we also measured the starting *C*_t_ value each time we thawed our viral stocks for experiments (see Materials and methods section and Supplementary Table S4)_._ Viral particles were incubated with cells for two hours to allow sufficient binding prior to washing. RT-PCR targeting *ORF1b*, *E*, and *N* genes was used to detect the presence of SARS-CoV-2 (see Materials and methods section). Cycle threshold values, *C*_t_, were used to quantify the viral titers, with lower values indicating higher viral titers. We included supernatant because cell death releases viral particles. Because the results from RT-PCR based on all the three targeted genes are highly concordant across all experiments in this study (*R* > 0.99, *P* < 2.2e^−16^; Supplementary Table S4), we will only discuss the results of the *ORF1b* gene (Fig. 3a). We failed to detect any significant signals from our negative controls, hence we simply assigned a *C*_t_ value of 40 (detection limit of the equipment).

Briefly, the *C*_t_ values of samples remained mostly flat with small fluctuations for all of the viral isolates at 1, 2, and 4 h P.I. (Fig. 3a and Supplementary Fig. S4a–d). During these early hours, viral particles bind to gain access into cells, and therefore replication rarely occurs^28^. At 8 h P.I., we observed significant decreases in *C*_t_ values (indicating increases in viral titers in cell culture) for most of the viral isolates (Fig. 3a and Supplementary Fig. S4d). At 24 h P.I., we observed significant decreases in *C*_t_ values for all of the viral isolates except for ZJU-2, although some of the viral isolates, namely ZJU-1, ZJU-7, ZJU-10, and ZJU-11, decreased much faster than the others (∆*C*_t_ ≥ 5; Fig. 3a, b and Supplementary Fig. S4d). The *C*_t_ values continued to decrease in all infected cells until the end of the experiment. Notably, at 24 h P.I., ZJU-3, ZJU-2, and ZJU-8, the latter two being members of the ORF8-L84S cluster (the majority of USA WA-Seattle isolates are in this group), showed considerably lower viral titers (Fig. 3b). We also observed a nearly 214-fold (2^7.74^) difference in mean viral titers between ZJU-10 and ZJU-3 at 24 h P.I. (Fig. 3b). These differences are reproducible between biological replicates (*R* = 0.927, *P* < 2.2e^−16^; Supplementary Fig. S4a–c). In summary, different viral isolates exhibit significant variation in replication dynamics when infecting Vero cells.

### Patient-derived SARS-CoV-2 isolates show consistent variations in replication dynamics when infecting human lung, liver, and kidney cancer cell lines

We next tested if the differences in viral replication dynamics can be reproduced in human cell lines, which are more clinically relevant. We chose the human lung cancer cell line Calu-3, human liver cancer cell line Huh-7, and the human kidney cancer cell line 293T, since the source organs of these cell lines have been implicated in SARS-CoV-2 infection^8–10^. To evaluate the best experimental conditions in different cell lines, we first infected different cell lines with ZJU-8 and ZJU-10 at different MOIs (1, 2, and 4). Viral isolates ZJU-8 and ZJU-10 were used because they represent the weaker and the stronger versions of SARS-CoV-2 based on the results in the Vero cell line. We observed that ZJU-10 consistently outperforms ZJU-8 in all cell line-MOI combinations (Fig. 3c–e and Supplementary Fig. S4e). Although higher MOIs did have an impact on the viral copy numbers, especially at earlier timepoints, different MOIs did not modify the viral replication dynamics in different cell lines (Fig. 3c–e and Supplementary Fig. S4e).

**Fig. 4:**
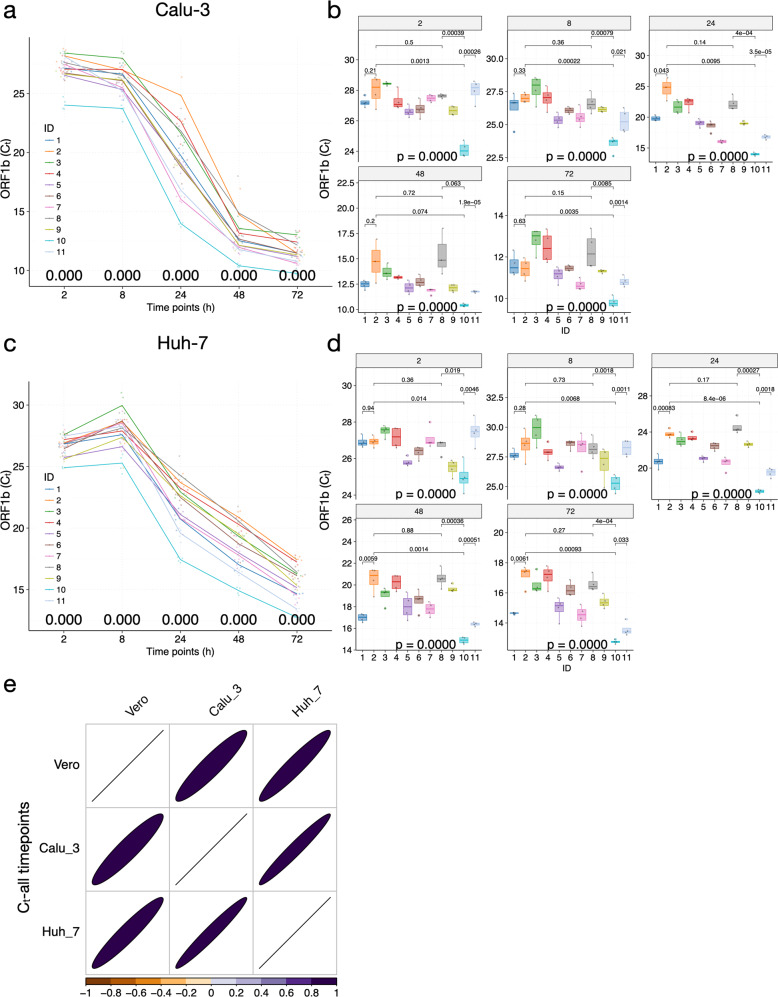
Patient-derived mutations in SARS-CoV-2 directly impact the viral replication dynamics in Calu-3 and Huh-7 cells. **a**–**d** Time-series plots of the *C*_t_ values (corresponding to the multiplicative inverse of viral titer) of the SAR-CoV-2 *ORF1b* gene over the course of infection in Calu-3 (**a**) and Huh-7 (**c**) cells. Significant variations in viral titer were observed at each timepoint in Calu-3 (**b**) and Huh-7 (**d**) cells. **e** Variation patterns of replication dynamics of the 11 viral isolates are highly consistent in Vero, Calu-3, and Huh-7 cell lines. The correlation coefficients were color-coded according to the bottom legend and also visualized in ellipses, with the circularity inversely related to the correlation coefficient; only correlation coefficients with adjusted *P*-values < 0.05 were shown. For all plots, each viral isolate was color-coded accordingly. At each timepoint, a *P*-value was calculated using the ANOVA method to compare the means of *C*_t_ values of different viral isolates. Pairwise *P*-values were calculated using the *t*-test and adjusted *P*-values are shown.

To preserve the original viral stocks, we replicated the experiments using MOI = 1 at 2, 8, 24, 48, and 72 h P.I. in the three human cancer cell lines. Strikingly, in Calu-3 and Huh-7 cell lines, the variations in replication dynamics among viral isolates are very well reproduced (*R* > 0.9 and *P* < 2.2e^−16^ across all comparisons; Figs. 3a, b, 4 and Supplementary Fig. S5a). Specifically, ZJU-7, ZJU-10, and ZJU-11 have consistently lower *C*_t_ at 24 h P.I. and beyond (Fig. 4a–d and Supplementary Fig. S5b, c). For both Calu-3 and Huh-7 cell lines, the biggest differences in viral titers among isolates were observed at 24 h P.I., with a staggering 1500-fold (2^10.6^) difference between ZJU-2 and ZJU-10 in the Calu-3 cell line and a 140-fold (2^7.16^) difference between ZJU-8 and ZJU-10 in the Huh-7 cell line (Fig. 4b, d). In the 293T cell line, probably due to the lack of innate SARS-CoV-2 binding receptors^29^, only a small decrease in *C*_t_ was observed in cells infected by ZJU-10 and later in cells infected by ZJU-7, although the differences are much subtler when compared to Huh-7 and Calu-3 cell lines (Supplementary Fig. S5d, e). Therefore, we conclude that the replication dynamics of different viral isolates remain consistent across different cell lines tested, except for 293T, in which the virus did not reproduce effectively (Fig. 4e and Supplementary Fig. S5a). Specifically, starting from 24 h P.I., the ZJU-7, ZJU-10, and ZJU-11 viral isolates show consistently higher viral titers, up to a 1500-fold difference between the extremities in the Calu-3 cell line.

### Patient-derived SARS-CoV-2 isolates show consistent variations in infectivity in Vero, Calu-3, and Huh-7 cell lines

**Fig. 5:**
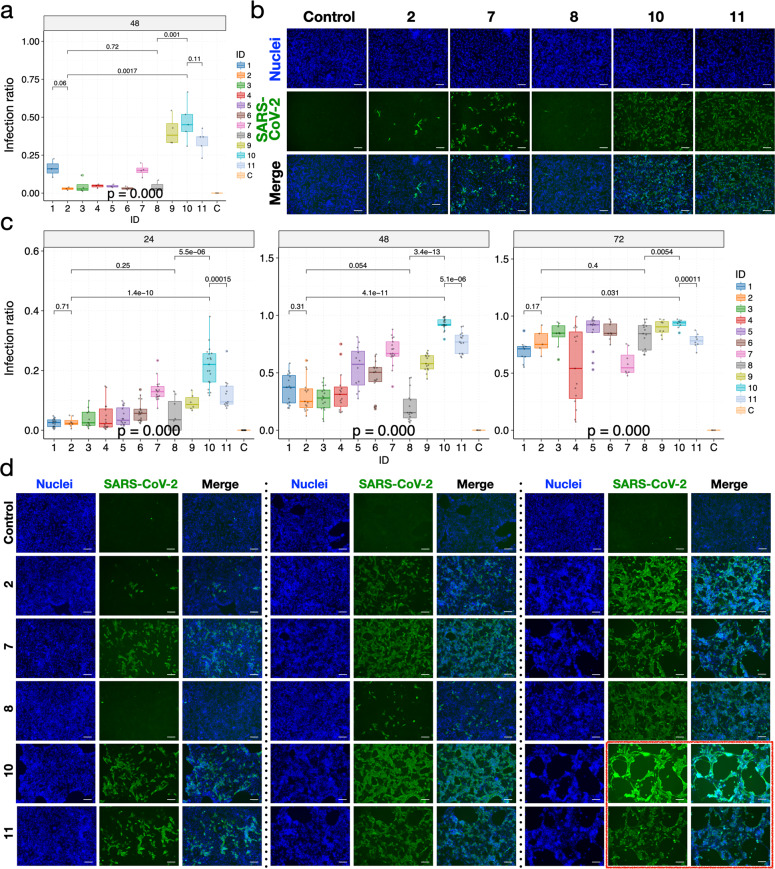
Patient-derived mutations in SARS-CoV-2 directly impact the viral infection ratios in Vero and Calu-3 cells. **a** Significant variations in infection ratio among viral isolates were observed at 48 h P.I. in Vero cells. **b** Representative fluorescent images of Vero cells (blue) infected by viral isolates (green; viral ID listed at the top). **c** Significant variations in infection ratio among viral isolates were observed across timepoints in Calu-3 cells. **d** Representative fluorescent images of Calu-3 cells (blue) infected by respective viral isolates (green; viral ID listed on the left). The red box indicates cells suffering from significant cytotoxic effects as a result of viral infections. For all plots, each viral isolate was color-coded accordingly. At each timepoint, a *P*-value was calculated using the ANOVA method to compare the means of *C*_t_ values of different viral isolates. Pairwise *P*-values were calculated using the *t*-test and adjusted *P*-values are shown. Scale bars, 100 µm.

**Fig. 6:**
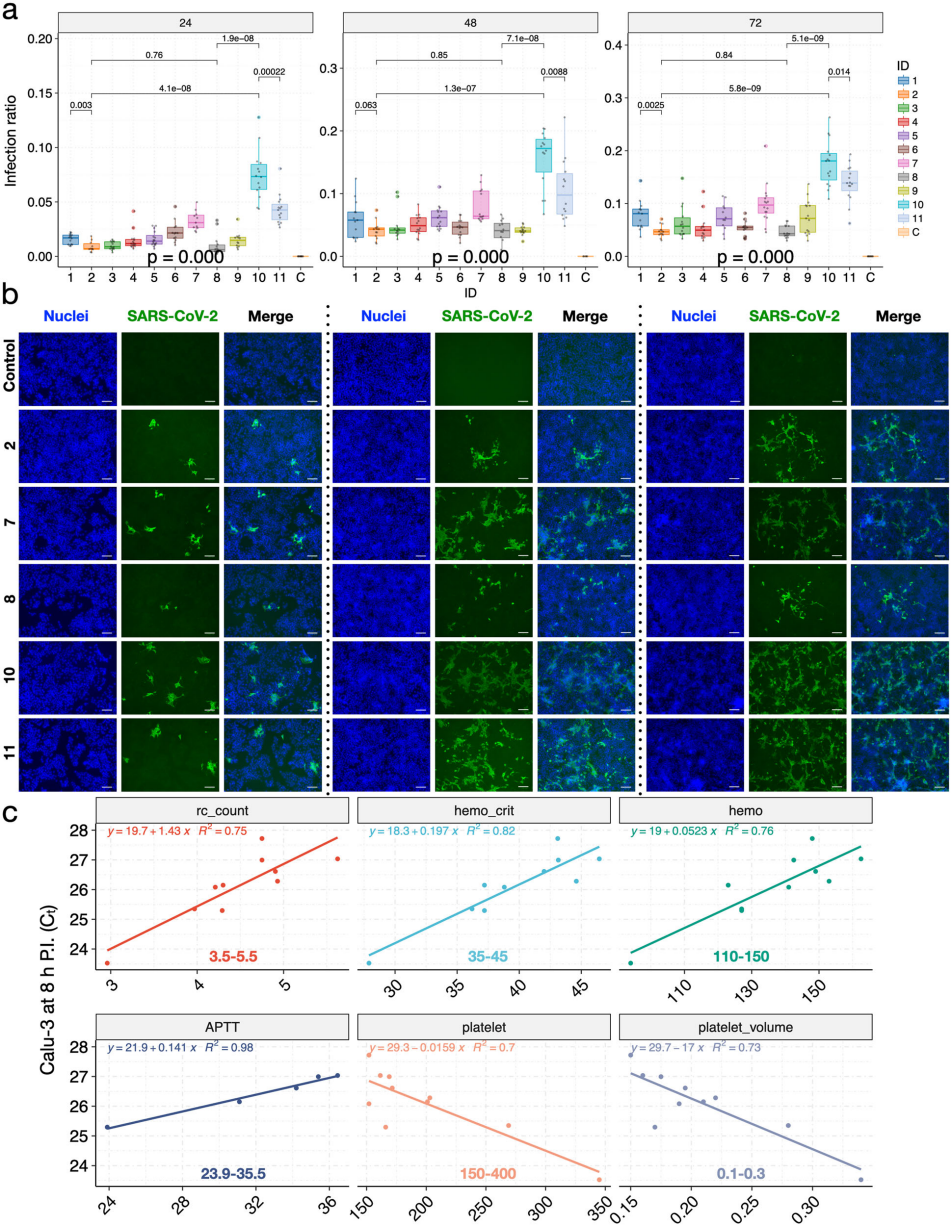
Patient-derived mutations in SARS-CoV-2 directly impact the viral infection ratios in Huh-7 cells. **a** Significant variations in infection ratio among viral isolates were observed across timepoints in Huh-7 cells. **b** Representative fluorescent images of Huh-7 cells (blue) infected by respective viral isolates (green; viral ID listed on the left). **c** Variations in viral titers correlated with the variations in patients’ clinical data. Regression functions and the normal ranges of each clinical variable (for women) are shown on each panel; units omitted for consistency. At each timepoint, a *P*-value was calculated using the ANOVA method to compare the means of *C*_t_ values of different viral isolates. Pairwise *P*-values were calculated using the *t*-test and adjusted *P*-values are shown. Scale bars, 100 µm.

Next, we investigated whether the higher viral titers correspond to more cells being infected in the culture. We performed immunofluorescent staining using antibodies against the SARS-CoV-2 S protein in different cell lines to reveal the cellular presence of the viral particles (in quadruplicates; see Materials and methods section). Overall, for different viral isolates, the variation pattern of infection ratios and viral titers was highly concordant (Figs. 5a–d, 6a, b and Supplementary Fig. S5a). Specifically, infection by viral isolates ZJU-10 and ZJU-11 consistently led to the highest infection ratios in the Vero, Calu-3, and Huh-7 cell lines (Figs. 5, 6 and Supplementary Fig. S6a, b), with ZJU-10 approaching a mean of 92.6% infection ratio at 48 h P.I. in the Calu-3 cell line (Fig. 5c, d, middle column). Notably, although the biggest differences in *C*_t_ among different viral isolates were seen at 24 h P.I. (Figs. 3a, b and 4a–d), their impact on the infection ratio was observed later, at 48 h P.I. (Figs. 5a, c and 6a). This is logical as the viruses would need to replicate to a sufficient number to be able to infect more cells.

Notably, there is a significant discrepancy in the general infection efficiency among cell lines. For example, at 48 h P.I., ZJU-10 had already infected around 40% and 92.6% of Vero and Calu-3 cell lines, respectively, but managed to infect < 20% of Huh-7 cells (Figs. 5a, c and 6a). In fact, the high infection ratios achieved by ZJU-10 and ZJU-11 in Calu-3 cells had already plateaued around 48 h P.I. and did not further increase at 72 h P.I., while other viral isolates caught up (Fig. 5c). However, significant viral cytotoxicity was observed for ZJU-10 and ZJU-11 when infecting Calu-3 cells at 72 h P.I., but not in Huh-7 (Fig. 5d, red box, 6b and Supplementary Fig. S6e, f). Finally, the infection ratios in all three cell lines, albeit with different infection efficiencies, show highly consistent variation patterns (Supplementary Figs. S5a, S7a; due to the saturation of infection in Calu-3 cells at 72 h P.I., data from that timepoint were not included in this analysis). Taken together, we demonstrated that the mutations in patient-derived viral isolates not only led to drastic differences in viral copy number but also resulted in corresponding drastic changes in infection ratio, especially in the Calu-3 cells, which led to significant cytotoxic effects.

### Variations in the viral titers of patient-derived SARS-CoV-2 isolates correlate with variations in patients’ clinical indices

To explore the potential clinical implications of the mutations, we collected 57 clinical examination indices of the 11 patients during patients’ stay at the hospital (Supplementary Tables S1 andS4). We focused on the clinical data around the viral sampling date (±1 day), as we believe the viral populations are likely evolving over time in vivo. Interestingly, the red blood cell count (rc_count), hemoglobin (hemo), the volume percentage of red blood cells (hemo_crit), and albumin levels were positively correlated with the *C*_t_ values of *ORF1b* in Calu-3 at 24 h P.I. (Supplementary Fig. S7b). The positive correlations between the red blood cell function and the *C*_t_ values of *ORF1b* remained consistent at 2 and 8 h P.I., but not at other timepoints (Fig. 6c and Supplementary Table S4), indicating that red blood cell function was impaired in patients infected by more potent viral isolates (lower *C*_t_ values). On the other hand, the blood clotting function appeared to be enhanced in patients infected by more potent viral isolates (based on *C*_t_ values in Calu-3 at 2 and 8 h P.I.; Fig. 6c and Supplementary Table S4). Specifically, while activated partial thromboplastin time (APTT) decreased in patients infected by more potent viral isolates, the level of platelet and platelet volume actually increased in patients infected by more potent viral isolates. In addition, the measurements of significantly correlated clinical indices, except for platelet, were out of the normal ranges in the ZJU-10 patient (Fig. 6c and Supplementary Fig. S7b). Taken together, the higher titers and infectivity of the ZJU-10 viral isolate, as measured in vitro, could directly impact the clinical conditions of ZJU-10 patients.

## Discussion

The quickly-developing COVID-19 pandemic has already infected more than 33 million victims and caused more than a million deaths globally. In this study, we sought to establish the genotype–phenotype link behind the abundant diversity being observed as a result of global sequencing efforts (GISAID and NCBI). Due to the abundant confounding factors in cohorts of patients and insufficient genomic sequencing of viruses from diagnosed patients, establishing a link between genotype and the clinical symptoms in patients is challenging. The in vitro cell line model provides an ideal system to quickly examine the impact of mutations in different isolates of the virus, where all patient-related confounding factors are removed. We first assessed the impact of mutations on viral replication dynamics and infectivity in the Vero cell line, then reproduced the findings in the human lung cancer cell line Calu-3, human liver cancer cell line Huh-7, and to a smaller extent, human kidney cancer cell line 293T. Several key findings stand out in our study:

First, a diverse collection of 46 mutations was identified in the 11 viral isolates. 21 of the 46 identified mutations are non-consensus mutations (frequency ≤ 50%). Even mutations with frequencies as low as 1% had hundreds of supporting reads due to our ultra-deep sequencing strategy. Intriguingly, the T22303G and A22301C mutations resulted in the same S247R mutation in the S protein (Fig. 1 and Supplementary Fig. S1). Mapping to the existing protein structure revealed that this residue is located in a flexible loop region within the N-terminal domain of the S1 subunit of the S protein, although the exact position of S247 could not be determined (Supplementary Fig. S7c–f, red arch). Although the N-terminal domain is not directly involved in binding to ACE2^7^, this domain is positioned right next to the C-terminal domain, which binds ACE2. The non-consensus mutations presumably represent evolutionary changes before fixation, as a result of either selective sweep or genetic drift^30^. For example, the T22303G mutation was observed in five viral isolates, albeit in very different frequencies (from 8.98% to 99.99%, Supplementary Table S3), indicating that this specific mutation was already present and even fixed in a significant number of people during the early spread, despite the fact that it is still largely missing from the current GISAID collection (only 3 of 52,552 deposited sequences had the mutation). It is likely that the T22303G mutation was not transmitted out of China during the early days.

Second, intriguingly, although ZJU-10 and ZJU-11 consistently perform better than other isolates, their means of achieving higher viral titers and infectivity may be different. Specifically, ZJU-10 consistently had a lower *C*_t_ value (higher titers) than the rest of the group starting from 2 h P.I. (Figs. 3b, 4b, d and Supplementary Fig. S5d, e) across all four cell lines. Because all excess viral particles were washed off at 2 h P.I. (see Materials and methods section), this observation indicates that the binding efficiency of ZJU-10 may be superior to its peers. In contrast, ZJU-11 typically has one of the highest, if not the highest, *C*_t_ values (lowest viral titers) at 2 h P.I. across different cell lines (Figs. 3b and 4b, d). Notwithstanding, ZJU-11 achieved significantly higher viral titers around 8 to 24 h P.I., indicating that its reproduction efficiency is likely the main driving force behind its overperformance among the isolates. Recent studies on the S-D614G mutation suggested that mutations at the S1/S2 sub-units interface could potentially enhance the binding of SARS-CoV-2 in vitro^16,18,31^. ZJU-10 has four missense mutations in the S protein, although only one of them is a consensus mutation (frequency ≥ 50%). These mutations could potentially contribute to its early stronger binding suggested by our study. On the other hand, ZJU-11 has a trinucleotide missense mutation and a stop-codon mutation (only at 6.56%) in the *ORF7b* gene, and its T22303G mutation is shared with several other isolates which do not show higher viral titers. The ORF7b protein is only 44 amino acids in length and highly hydrophobic. ORF7b is speculated to be a transmembrane protein and possibly a viral structural protein. The two identified amino acid changes in the *ORF7b* gene, asparagine, and threonine, are polar and could potentially disrupt the hydrophobic nature of this protein. Interestingly, the patient carrying ZJU-11 remained positive for an extended period of 45 days (Supplementary Table S1). In the current GISAID database, another trinucleotide mutation (G28881A, G28882A, and G28883C), which is shared by thousands of viral isolates, also results in two missense mutations at the protein level (Supplementary Fig. S8). Given the complicated nature of multiple mutations in each viral isolate, the detailed mechanisms of how these viral isolates achieved higher viral titers would need extensive further studies.

Third, our finding that variations in viral titers of different SARS-CoV-2 isolates were positively correlated with blood clotting function but inversely correlated with red blood cell function and the level of albumin is very intriguing. It has been estimated that at least 1/3 of the COVID-19 patients developed serious blood clotting issues^32^. Significant blood vessel damages and micro blood clots were found in the lungs of COVID-19 patients^33^. The ACE2 receptor is abundant in blood vessels, therefore, SARS-CoV-2 may directly attack blood vessels in patients and induce blood clotting^34^. Furthermore, anti-blood clotting treatments were shown to be effective in prolonging the median survival time of patients^35^. We hypothesize that the more potent viral isolates could inflict more damage on the blood vessels, impair the red blood cell function, and induce stronger blood clotting function in patients, potentially leading to a worse clinical outcome. Although the ZJU-10 patient did not show more critical symptoms during the hospital stay, several clinical indices of ZJU-10 were out of the normal ranges. More data are needed to confirm the relationship between viral potency and blood vessel damages in patients.

Forth, in contrast to a recent report that a viable viral isolate could not be obtained from stool samples^36^, three of our viral isolates were extracted from stool samples (two of which were very potent) indicating that viable SARS-CoV-2 particles could be found in stool samples. Furthermore, the detailed time-series data generated in our study involving the use of multiple viral isolates and human cell lines could be informative to researchers who seek to study the functional aspects of SARS-CoV-2 in different tissue types (Supplementary Tables S3 and S4). Specifically, we consider the Calu-3 cell line to be a great experimental system.

In short, our study provides direct evidence that mutations currently occurring in the SARS-CoV-2 genome can drastically impact viral replication dynamics and infectivity within 24–48 h P.I. across multiple cell lines, including the human lung cancer cell line Calu-3. The correlative results between the viral titers and various clinical indices in patients suggest that mutations observed in SARS-CoV-2 have the potential to impact viral pathogenicity through blood vessel-related damages. Therefore, viral mutations should not only be monitored through sequencing, but also be investigated in detail when possible. Functional characterizations of naturally occurring mutations of SARS-CoV-2 could be very useful in designing strategies to fight against the virus and understanding the evolution of the virus to prevent the next pandemic.

## Materials and methods

### Experimental model and subject details

Patients with confirmed COVID-19 were admitted to the First Affiliated Hospital from Jan 19 to Mar 5, 2020. The First Affiliated Hospital, located in Hangzhou, Zhejiang Province, China, is one of the major provincial hospitals designated to receive patients with COVID-19 infection across the Zhejiang Province; therefore, patients with severe symptoms outside of Hangzhou were also admitted. Starting from Jan 10, 2020, all patients presenting to the hospital’s fever clinic were screened by clinical staff for COVID-19 infection utilizing criteria for suspected cases as defined by the National Health Commission of China’s clinical diagnosis and management guideline for COVID-19^37^. Briefly, patients were screened based on their clinical symptoms and their risk of epidemiological exposure, including past travel to Hubei Province or close contact with people who had visited Hubei Province during the COVID-19 outbreak. As the pandemic continued to spread, the probability of transmission outside of Hubei Province increased. The epidemiological exposure to Hubei Province was not a prerequisite for suspected cases. All suspected cases were determined by laboratory tests and based on positive results of a qRT-PCR assay for COVID-19. Patients were excluded if two qRT-PCR tests 24 h apart both suggested negative results. Patients’ clinical samples with *C*_t_ ≤ 28 were collected to isolate SARS-CoV-2. Data of 57 clinical features during the course of treatment were collected from affiliated hospitals.

### Method details

#### Cell lines and culturing conditions

The Vero (African green monkey kidney, ATCC^®^ CCL-81^™^) cell line, Calu-3 (Human lung adenocarcinoma, ATCC^®^ HTB-55^™^) cell line, 293T (Human embryonic kidney, ATCC^®^ CRL-3216^™^) cell line, and Huh-7 (Human hepatocellular carcinoma, Japanese Collection of Research Bioresources, JCRB0403) cell line were grown in Dulbecco’s Modified Eagle’s Medium (DMEM; Gibco BRL, Grand Island, NY, USA) supplemented with 10% fetal bovine serum (FBS; Gibco BRL). The cells were maintained in a 5% CO_2_ incubator at 37 °C. Cells were harvested and passaged after treatment with 0.25% trypsin and 0.02% EDTA.

For all experiments in this study, cells were counted by the Vi-Cell XR (Beckman Coulter, Miami, FL, USA) and seeded at a density of 1 × 10^5^ cells/well in 24-well plates 1 day (for Vero, Huh-7, and 293T cells) or 2 days (for Calu-3 cells) prior to viral infection to reach 80%–90% confluency.

#### Sample collection, virus isolation, cell infection, and electron microscopy

The study was approved by the Clinical Research Ethics Committee of The First Affiliated Hospital, School of Medicine, Zhejiang University (Approval notice 2020-29) for emerging infectious diseases. All samples, sources including sputum, nasopharyngeal swab, and stool, were collected from COVID-19 patients with consent (Supplementary Table S1). All collected samples were sent to the biosafety level III laboratory for viral isolation within 4 h (biosafety level approved by China National Accreditation Service for Conformity Assessment (CNAS No. BL0022), State Key Laboratory for Diagnosis and Treatment of Infectious Diseases, the First Affiliated Hospital, School of Medicine, Zhejiang University).

The sputum, stool, and nasopharyngeal swab samples were pre-processed by first mixing to appropriate volume (Sputum, 5–10 volumes; Stool, 2 mL/100 mg; Nasopharyngeal swab, 1 volume), using DMEM medium with 2% FBS, Amphotericin B (100 ng/mL), Penicillin G (200 units/mL), Streptomycin (200 μg/mL), and TPCK-trypsin (4 μg/mL). The supernatant was collected after centrifugation at 1800 × *g* at room temperature (25 °C). Before infecting Vero cells, all collected supernatant was filtered using a 0.45 µm filter (Merck Millipore, Billerica, MA, USA) to remove solid debris, etc.

For viral isolation and infection, 3 mL of filtered supernatant of each sample was added to Vero cells in a T25 culture flask. After incubation at 35 °C for 2 h to allow binding, the inoculum was removed and replaced with 5 mL fresh culture medium. The cells were incubated at 35 °C and observed daily to evaluate cytopathic effects (CPE). The culturing supernatant was tested for SARS-CoV-2 by qRT-PCR (see below for qRT-PCR protocol). Once the qRT-PCR test shows positive results with *C*_t_ ≤ 25 (typically after 4–5 days of incubation), the live viral particles were collected from the culture supernatant by centrifugation (1800 × *g* for 20 min at 4 °C). The viral stocks were then aliquoted into cryotubes at 0.2 mL/tube and stored at −80 °C. The viral stocks were used for all experiments in this study.

For transmission electron microscopy (TEM), the viral cultures were inactivated with 4% paraformaldehyde for 48 h at 4 °C and centrifuged at 100,000 × *g* for 2 h at 4 °C. The viral particles were observed with a Tecnai G2 transmission electron microscope (Philips Electronic Instruments Co., The Netherlands) at 200 kV.

#### Estimation of TCID_50_ and titers of the viral stocks

The titers of the patient-derived viral stocks were estimated using the standard TCID_50_ method as previously described^38^. PFUs/mL of each viral stock was calculated by multiplying the TCID_50_ with the empirical coefficient 0.7 as recommended by the American Type Culture Collection (ATCC). The MOI was then calculated based on the number of cells plated in each well (1 × 10^5^) in the experiments.

#### Viral replication dynamics assay

Cells in 24-well plates were infected with different SARS-CoV-2 isolates in quadruplicates at 0.5 MOI (Vero cells) or 1 MOI (Calu-3, Huh-7, and 293T cells). The inoculum was removed at 1 h P.I. for the 1-h timepoint group and at 2 h P.I. for other timepoint groups. After incubation for 1 or 2 h, the cells were washed with PBS three times and replete with 1 mL fresh medium to remove non-binding viruses. The cell culture plates were frozen immediately at −80 °C for the 1- and 2-h samples, or continued to grow for the other groups (4, 8, 24, 48, and 72 h) before frozen at −80 °C. Finally, all frozen samples from each timepoint were thawed and homogenized with pipetting and subjected to automatic nucleic acid extraction (EX3600; Liferiver Biotech, Shanghai, China) with a magnetic beads-based kit (Cat No. MVR01; Liferiver Biotech). Briefly, an aliquot of 200 μL homogenized culture was added to the sample well of the preassembled plate, followed with the addition of 20 μL proteinase K. Then the plate was loaded to the automatic nucleic acid extraction machine. Through a series of steps including sample lysis, nucleic acid binding, and washing, purified nucleic acids were isolated and collected, and the viral nucleic acid abundance was measured using SARS-CoV-2 qRT-PCR kits (Liferiver Biotech), targeting *ORF1b*, *E*, and *N* genes. Results from the first two timepoints (1 and 2 h P.I.) reflected the ability of viral attachment to cells, and results from the later timepoints represented the viral replication dynamics. Cells collected at 24, 48, and 72 h were used for immunofluorescence staining without freezing.

#### Immunofluorescence staining

Cells grown in monolayers under different conditions were fixed in 80% acetone (chilled at −20 °C) at 4 °C for 20 min. The cells were then washed three times with ice-cold PBS, blocked with 1% bovine serum albumin (BSA) for 30 min, and incubated with anti-SARS-CoV-2 Spike-RBD rabbit monoclonal antibody (diluted in PBS with 0.1% BSA at 1:1000; Sino Biological Inc, Beijing, China) at 4 °C overnight. The cells were again washed three times with ice-cold PBS and then stained with the Alexa Fluor488®-conjugated Goat Anti-rabbit IgG secondary antibody (diluted in PBS with 0.1% BSA at 1:1500; Invitrogen, Carlsbad, CA, USA) for 1 h at room temperature in the dark. The cells were washed three times and then incubated with 0.5 μg/mL 4′,6-diamidino-2-phenylindole (DAPI, nuclear DNA staining; Solarbio Life Sciences, Beijing, China) for 5 min. The cells were washed three times; immunofluorescence pictures were captured using the Bio-Rad ZOE Cell Imager (Hercules, CA, USA) equipped with a fluorescence apparatus.

#### Sequencing library construction

The total RNA in each deactivated viral sample was extracted using a viral RNA mini kit (Qiagen, Germany). The sequencing library was constructed using the Kapa RNA HyperPrep kit (Kapa, Switzerland) and deep-sequenced on the Illumina Novaseq 6000 platform (2 × 151 bases; Illumina Inc., San Diego, CA) by BGI genomics.

### Quantification and statistical analysis

#### Statistical analyses and visualization

The majority of statistical analyses and visualizations were done in Rstudio and R (at the time of writing, 1.0143 for Rstudio and 3.4.0 for R), with necessary aid from customized python scripts (2.7.4) and shell scripts (Linux). The primary R packages are mostly maintained by the Bioconductor project (https://www.bioconductor.org/, along with all their dependencies). The essential ones used are ggplot2 (2.2.1), reshape2 (1.4.3), RColorBrewer (1.1–2), scales (0.5.0), corrplot (0.84), Hmisc (4.1–1), ggrepel (0.7.0), cluster (2.0.6), factoextra (1.0.5), plyr (1.8.4), dplyr (0.7.4), psych (1.7.8), devtools (1.13.4), ggpubr (0.1.6), tidyverse (1.2.1), gridExtra (2.3), ggsci (2.8), ggbeeswarm (0.6.0), ggpmisc (0.2.16), and colorspace (1.3–2).

In general, parametric statistical tests (*t*-test, ANOVA, and Pearson correlation) were used when the data distribution conformed to a normal distribution (such as qRT-PCR measurements), and non-parametric statistical tests (Wilcoxon test, Kruskal–Wallis, and Spearman correlation) were used when datasets did not conform to the normality assumption. We adjusted the *P*-values using the Benjamini & Hochberg (BH) method^39^ to control for False Discovery Rate (FDR), when multiple comparisons were concerned, including the *P*-value matrix constructed when calculating the correlations matrix among different features or samples. The 3D structure of the S protein was visualized and downloaded from https://www.rcsb.org/3d-view/6VSB/1.

#### Sequence data processing and de novo assembling

Sequencing data were generated from Novaseq 6000 and first filtered of low-quality and high-barcode contamination by Soapnuke and then mapped to 43 complete genome references of 2019-nCoV (SARS-CoV-2) by BWA-MEM^40^. References of SARS-CoV-2 were downloaded from NCBI on date February 28th, 2020. Further, mapping reads that longer than 100 nt were extracted for de novo assembly by SPAdes^41^ (v3.1.3) using an iterative short-read genome assembly module for pair-end reads. *K*-values were selected automatically at 33, 55, and 77 nt for these samples. After assembling, contigs were blasted to nt database (20190301) to confirm their origins, and only contigs belonging to coronavirus were retained for base correction. Next, filtering reads of each sample were mapped back to retained assembled contigs and bam-readcount was applied (--min-mapping-quality = 5, other parameters were set as default) to calculate the base frequency of every post of each assemble contigs.

#### Single nucleotide polymorphism (SNP) analysis

To identify the SNPs in the patient-derived SARS-CoV-2 isolates, a customized SNP calling pipeline was developed. Briefly, Fastp (0.20.1) was used for quality control, filtering, and trimming of the raw reads. The cleaned reads were mapped against the SARS-CoV-2 reference genome (NC_045512.2) with bwa-mem (0.7.15-r1140). All mapped reads were extracted with samtools (1.6), and processed into mpileup files by samtools mpileup command. Calling of the variants was performed by VarScan^42^ (2.4.4) with parameters “--min-reads2 5 --min-var-freq 0.05 --strand-filter 0”. All mutations above 5% were characterized. The results were further inspected and confirmed manually using IGV (2.8.2).

#### Phylogenetic analysis

We acquired 725 high quality and high coverage SARS-CoV-2 genomes from GISAID (downloaded on 3/21/2020), including the Yunnan RaTG13 viral strain and the Guangdong pangolin viral strain as the outgroup. We chose to investigate the phylogenetic context of the viral isolates using data from the early phase of the pandemic because the later viral spread and exchange would mask the spatial-temporal signatures unique to this period. We aligned the 736 genomic sequences with MAFFT^43^ with options --thread 16 --globalpair --maxiterate 1000 and trimmed the full-length alignment with trimAL^44^ using the -automated1 option to remove any spurious parts of the alignment, which could introduce noise to the phylogenetic analysis process. We used iqtree^45^ with options -bb 1000 -alrt 1000 -nt 64 -asr to construct a 1000-times bootstrapped maximum-likelihood phylogenic tree of the 736 viral sequences based on 835 parsimony informative sites. The resulting phylogenetic tree was visualized in iTOL^46^. We conducted Tajima’s test of neutrality based on the constructed alignment of viral sequences using MEGA 7^47^.

#### Image analysis and infection ratio estimation

The SARS-CoV-2 infection ratios in different cell lines were quantitated using Fiji (Version: 2.0.0-rc-69/1.52p). For each sample, at least 8 immunofluorescence images were randomly taken for data analyses. For each immunofluorescence image, the infection ratio was calculated by dividing the number of SARS-CoV-2-infected cells (stained with SARS-CoV-2 Spike rabbit monoclonal antibody) by the total number of cells (DAPI-stained). The total number of cells (DAPI-stained) were quantitated as follows: select “Edit” – “Options” – “Conversions” to “scale when converting”; select “Image” – “Type” – “8-bit” to convert the image to greyscale; adjust the threshold to highlight all of the blue-stained areas; segment the few clustered cells with “Process” – “Binary” – “Watershed”; finally, count the total number of cells by “Analyze” – “Analyze Particles”. The SARS-Cov-2-infected cells were counted using “Process” – “Find Maxima” function and parameters were selected based on a randomly selected group of manually curated pictures (*n* = 15) for consistent results (manual vs automated, *R* > 0.85, *P* < 0.05).

## Supplementary information

Supplementary information

## Data Availability

The full-genome sequences of the 11 viral isolates have been deposited to the GISAID collection with the following IDs: EPI_ISL_415709, EPI_ISL_416042, EPI_ISL_416044, EPI_ISL_416046, EPI_ISL_415711, EPI_ISL_416047, EPI_ISL_416425, EPI_ISL_416473, EPI_ISL_416474, EPI_ISL_418990, and EPI_ISL_418991. The raw sequencing reads were submitted to NCBI under the BioProject ID PRJNA643359.

## Supplementary information

**Supplementary Information** accompanies the paper at (10.1038/s41421-020-00226-1).
