# Supplementary material for: Patient-derived SARS-CoV-2 mutations impact viral replication dynamics and infectivity in vitro and with clinical implications in vivo
Source: Cell Discov. 2020 Oct 29;6:76. doi: 10.1038/s41421-020-00226-1 (PMC7595057; doi:10.1038/s41421-020-00226-1)
Supplement: Supplementary file 1 — Supplementary information [file 41421_2020_226_MOESM1_ESM.pdf]

Supplementary table S1. A summary of the epidemiological information of the 11 patients involved in this study. The "Viral gen" (viral generation) was inferred based on their exposure history.

ID	Sex	Age	Sample	Sample collection date	Virus isolating date	Epide-miology	Viral gen	Onset sypmtoms	Severity	Blood presure	ICU	Onset date	Admission	Discharge
ZJU_1	M	30-40	Sputum	1/25	1/29	Contact with people from Wuhan	2	Fever	Moderate	0	0	1/23	1/25	2/23
ZJU_2	M	30-40	Sputum	1/26	1/29	Lived in Wuhan	1	Fever	Severe	0	0	1/23	1/24	2/23
ZJU_3	M	30-40	Sputum	1/25	1/30	Traveled to Wuhan	2	Fever	Severe	0	0	1/18	1/19	2/12
ZJU_4	M	30-40	Sputum	1/24	1/28	Conference with colleagues from Wuhan	2	Fever, fatigue	Moderate	0	0	1/17	1/21	2/9
ZJU_5	F	20-30	Sputum	1/22	1/26	Conference with colleagues from Wuhan	2	Fever	Severe	0	0	1/21	1/22	2/9
ZJU_6	M	70-80	Sputum	2/2	2/6	Contact with people who had COVID19 Wuhan.	2 or 3	Fever, fatigue	Severe	1	0	1/22	1/26	2/19
ZJU_7	F	0-1	Nasopharyngeal swab	2/3	2/8	Contact with people from Wuhan	2	Fever	Mild	0	0	1/29	1/29	2/20
ZJU_8	M	50-60	Sputum	1/26	1/30	Lived in Wuhan	1	Fever, fatigue	Critical	1	1	1/17	1/22	2/19
ZJU_9	M	30-40	Stool	1/28	2/7	Conference with colleagues from Wuhan	2	Fever	Severe	0	0	1/18	1/21	2/5
ZJU_10	F	30-40	Stool	2/3	2/8	Lived in Wuhan	1	Fever	Severe	0	0	1/19	1/27	2/12
ZJU_11	M	60-70	Stool	2/4	2/9	Lived in Wuhan	1	Coughing	Severe	1	0	1/19	1/26	3/15

Supplementary table S2. A summary of the sequencing statistics of the 11 viral isolates involved in the study, related to Figure 1.

ID	Raw reads	Clean reads	Raw bases(G)	Clean bases(G)	coverage	Clean rate	Error_rate_fq1	Error_rate_fq2
ZJU_1	239,755,367	227,931,734	71.93	63.96	2,138,916	88.92%	0.04%	0.04%
ZJU_2	211,974,282	195,211,677	63.59	53.28	1,781,761	83.78%	0.04%	0.04%
ZJU_3	421,726,717	378,718,257	126.52	103.91	3,474,902	82.13%	0.04%	0.04%
ZJU_4	485,306,221	434,439,201	145.59	118.99	3,979,199	81.73%	0.04%	0.04%
ZJU_5	232,311,525	205,222,721	69.69	56.11	1,876,400	80.52%	0.04%	0.04%
ZJU_6	342,183,998	273,708,578	102.66	70.92	2,371,668	69.09%	0.04%	0.04%
ZJU_7	227,769,540	191,916,976	68.33	52.18	1,744,975	76.37%	0.04%	0.05%
ZJU_8	355,648,629	331,651,060	106.69	90.7	3,033,140	85.01%	0.04%	0.04%
ZJU_9	287,524,792	260,634,803	86.26	72.7	2,431,194	84.28%	0.04%	0.04%
ZJU_10	136,595,606	101,832,137	40.98	28.24	944,387	68.91%	0.02%	0.03%
ZJU_11	121,791,338	96,729,474	36.54	27.76	928,335	75.98%	0.02%	0.03%
Average	278,417,092	245,272,420	83.53	67.16	2,245,898	79.70%	0.04%	0.04%

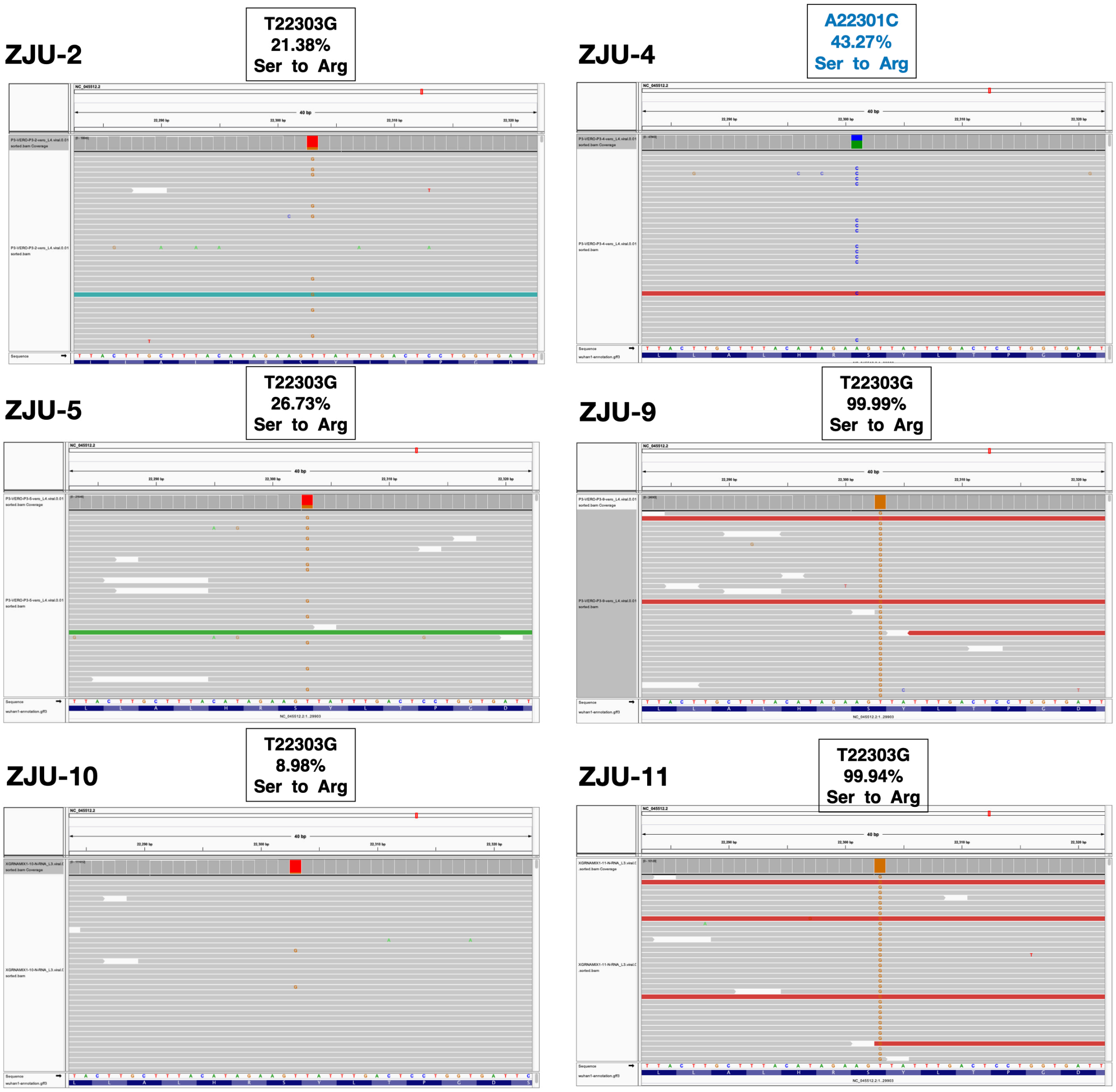

Supplementary figure S1. A summary of the nucleotide mutations that lead to the S247R mutations observed in the 11 patient-derived isolates, related to Figure 1. The mutation frequencies were shown. Images were produced by IGV.

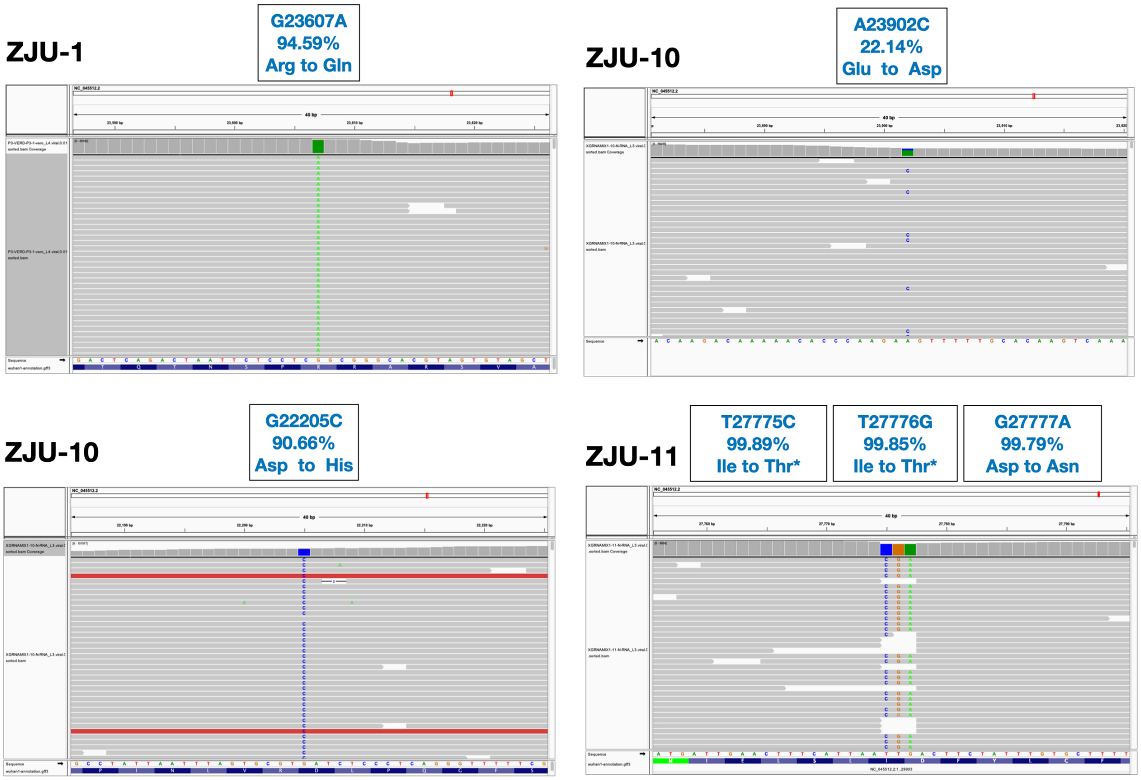

Supplementary figure S2. A summary of selected additional mutations in the S gene and the tri-nucleotide mutation, Related to Figure 1. Note that some of the mutations are in the form of minor alleles. Images were produced by IGV.

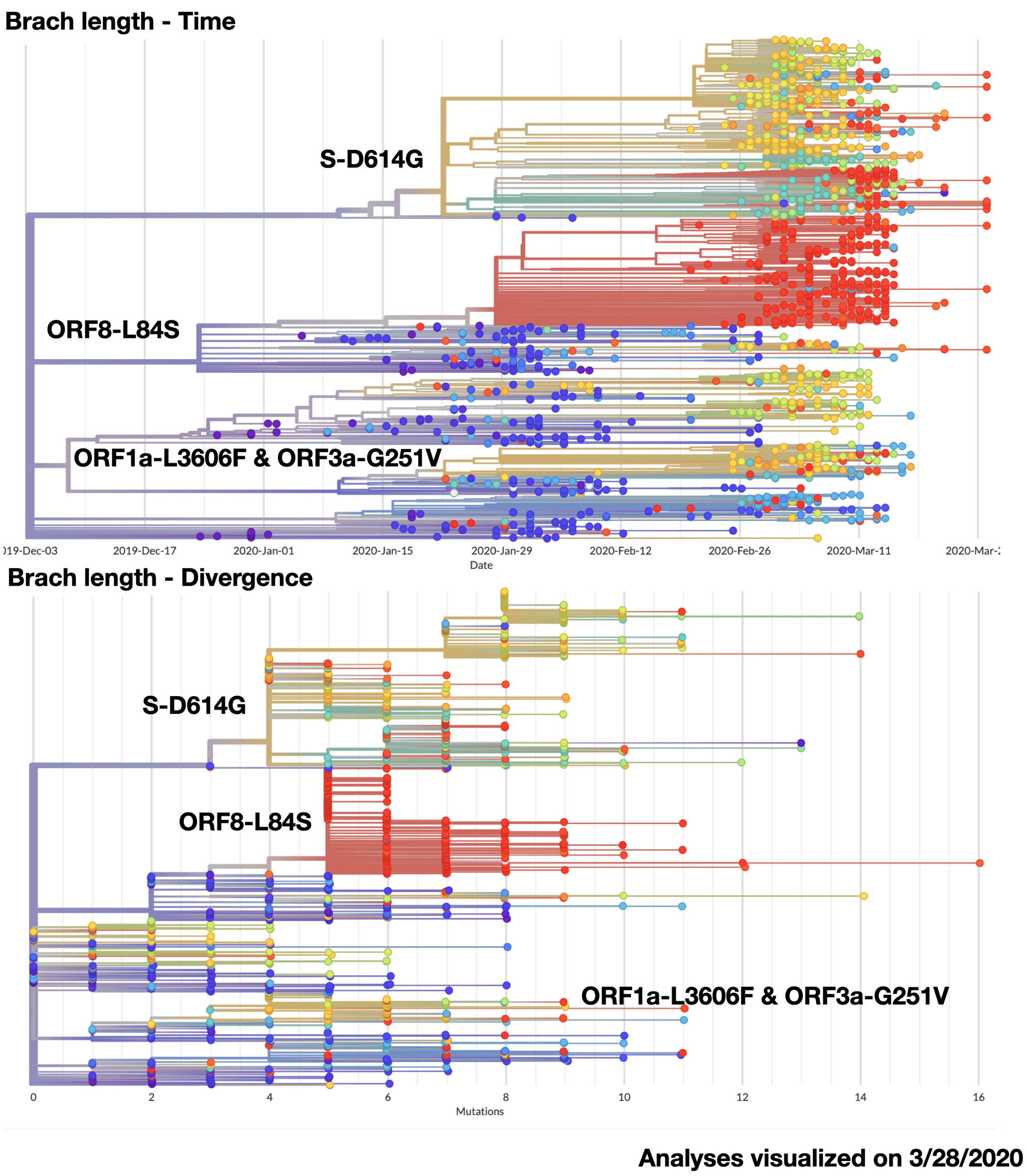

Supplementary figure S3. Phylogenetic analyses produced from GISAID using time (top) or number of mutations (bottom) as the branch length. Related to Figure 2. Note that all three major clusters described in the study are labeled accordingly. The major distinction

is that the ORF8-L84S clade is not monophyletic in our more computationally intensive and bootstrapping-supported approach.

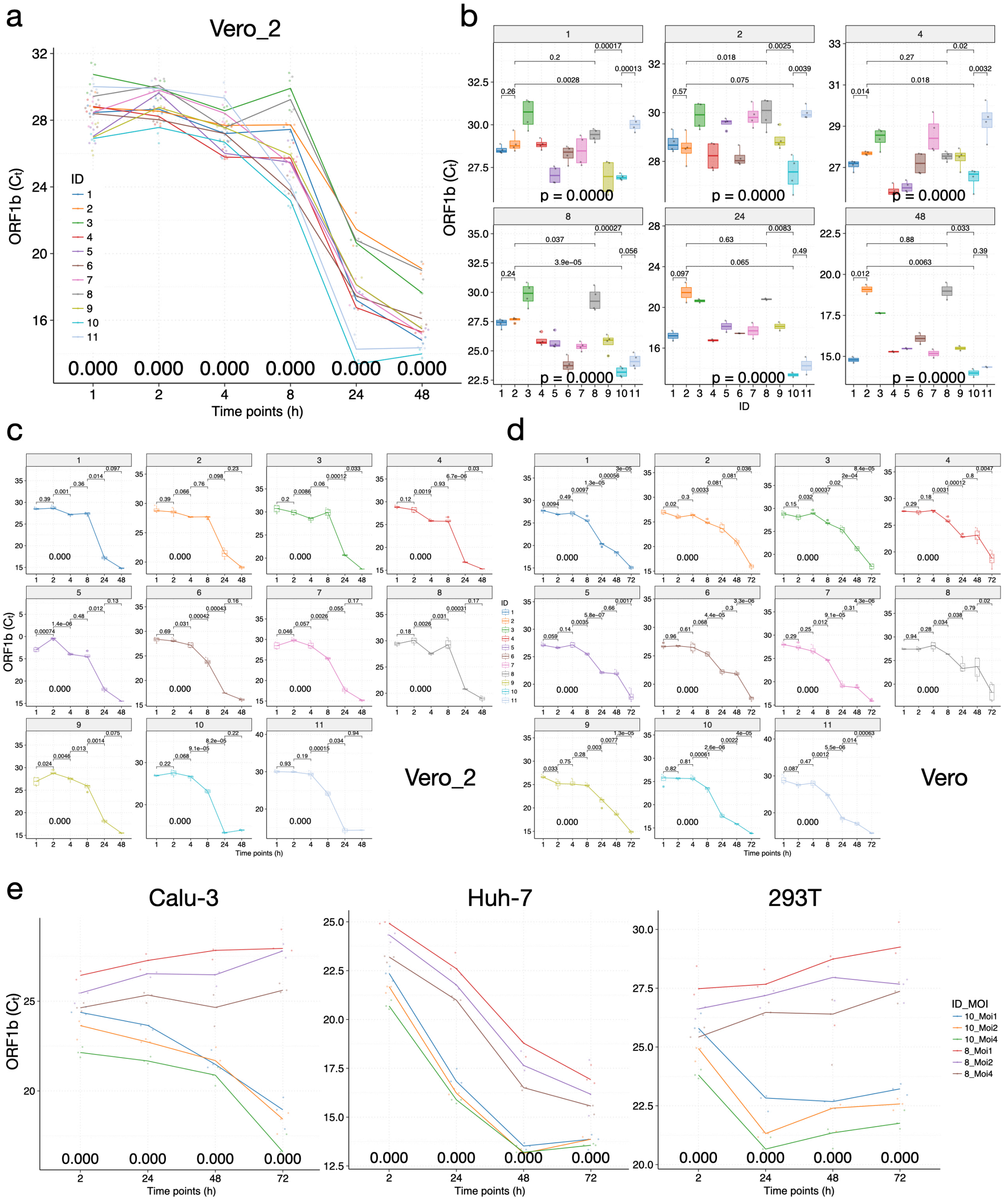
a
Vero_2
ORF1b (Ct)
ID
Time points (h)
b
p = 0.0000
ID
c
Vero_2
Time points (h)
d
Vero
e
Calu-3
Huh-7
293T
ID_MOI
10_Moi1
10_Moi2
10_Moi4
8_Moi1
8_Moi2
8_Moi4

Supplementary figure S4. The variations in viral titer among viral isolates are consistent in replicating experiments and different cell lines. (a) Time-series plots of the C_t values (corresponding to the multiplicative inverse of viral titer) of the SAR-CoV-2 ORF1b gene over the course of infection in Vero (replicating experiment). (b) Significant variations in viral titer were observed at each time point in Vero (biological replicate). (c) Time-series plots of the C_t values of the SAR-CoV-2 ORF1b for each of the 11 patient-derived viral isolates in Vero (biological replicate). (d) Time-series plots of the C_t values of the SAR-CoV-2 ORF1b for each of the 11 patient-derived viral isolates in Vero (related to Fig. 3a and b). (e) Time-series plots of the C_t values of the SAR-CoV-2 ORF1b for each MOI-cell line combination (related to Fig. 3c-e). For all plots, each viral isolate was color-coded accordingly. At each timepoint, a p-value was calculated using the ANOVA method to compare the means of C_t values of different viral isolates. Pair-wise p-values were calculated using the t-test and adjusted p-values are shown.

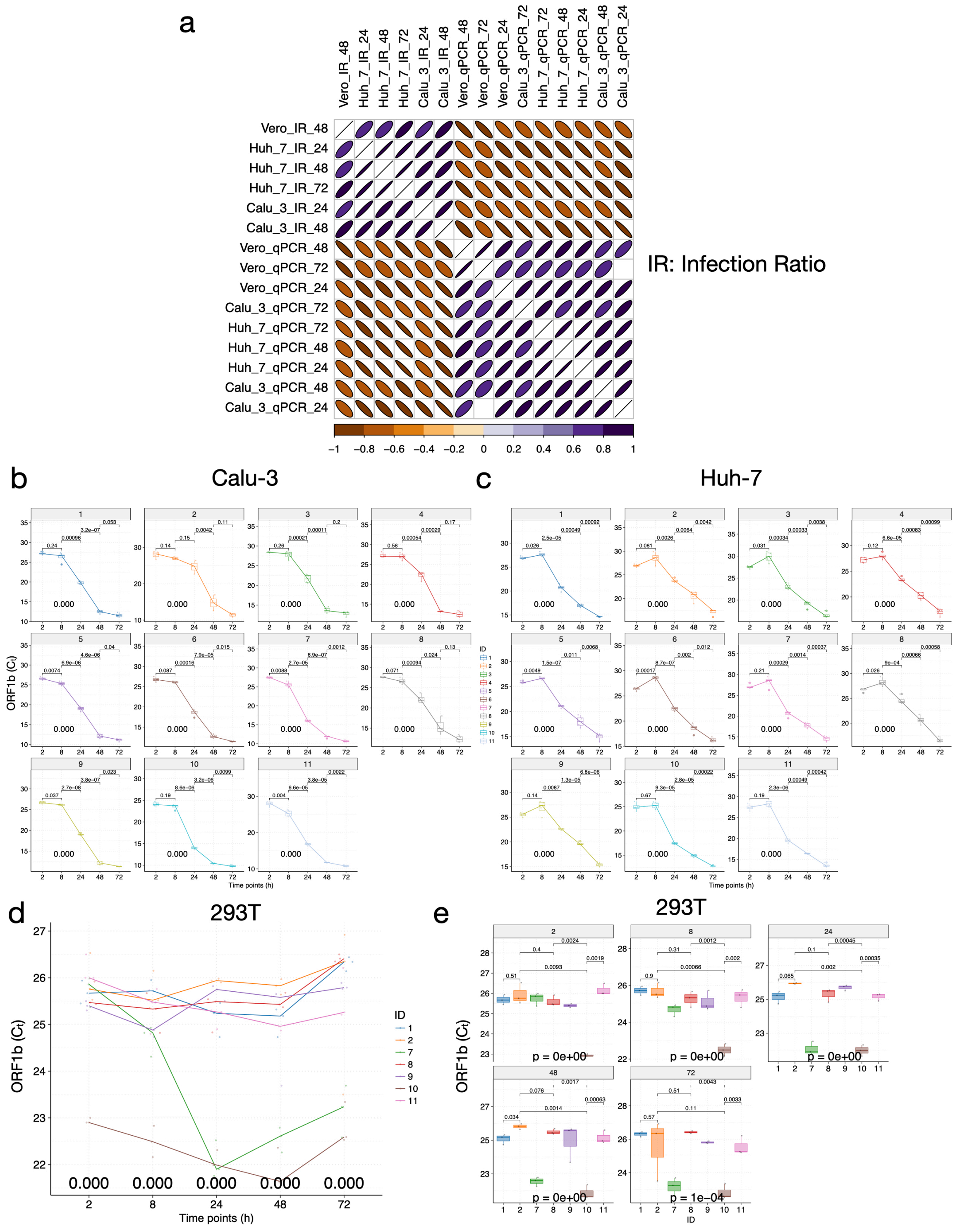

Supplementary figure S5. The variations in viral titer and infection ratio among viral isolates are consistent in different cell lines. (a) Variations in viral titer (measured in C_t

values) and infection ratio are highly correlated. Correlation coefficients between viral titers or infection ratios in different cell lines at different timepoints were calculated (timepoints are denoted by the numbers in column and row names). Note that due to the saturating infection in Calu-3 cell line after 48 hours P.I., we did not include data points from 72 hours P.I.. The viral titers are generally negatively correlated with infection ratios because they were measured in C_t values, corresponding to the multiplicative inverse of viral titers. The correlation coefficients are color-coded according to the bottom legend and also visualized in ellipses, with the circularity inversely related to the correlation coefficient; only correlation coefficients with adjusted p-values < 0.05 were shown. (b-c) Time-series plots of the C_t values of the SAR-CoV-2 ORF1b for each of the 11 patient-derived viral isolates in Calu-3 (b) and Huh-7 (c) cell line, related to Fig. 4a-d. (d-e) The time-series plot (d) and box plots (e) for selected viral isolates in 293T cell line. Note that except for ZJU-10 and ZJU-7, most viral isolates could not replicate in the 293T cell line. For all plots, each viral isolate was color-coded accordingly. At each timepoint, a p-value was calculated using the ANOVA method to compare the means of C_t values of different

viral isolates. Pair-wise p-values were calculated using the t-test and adjusted p-values are shown.

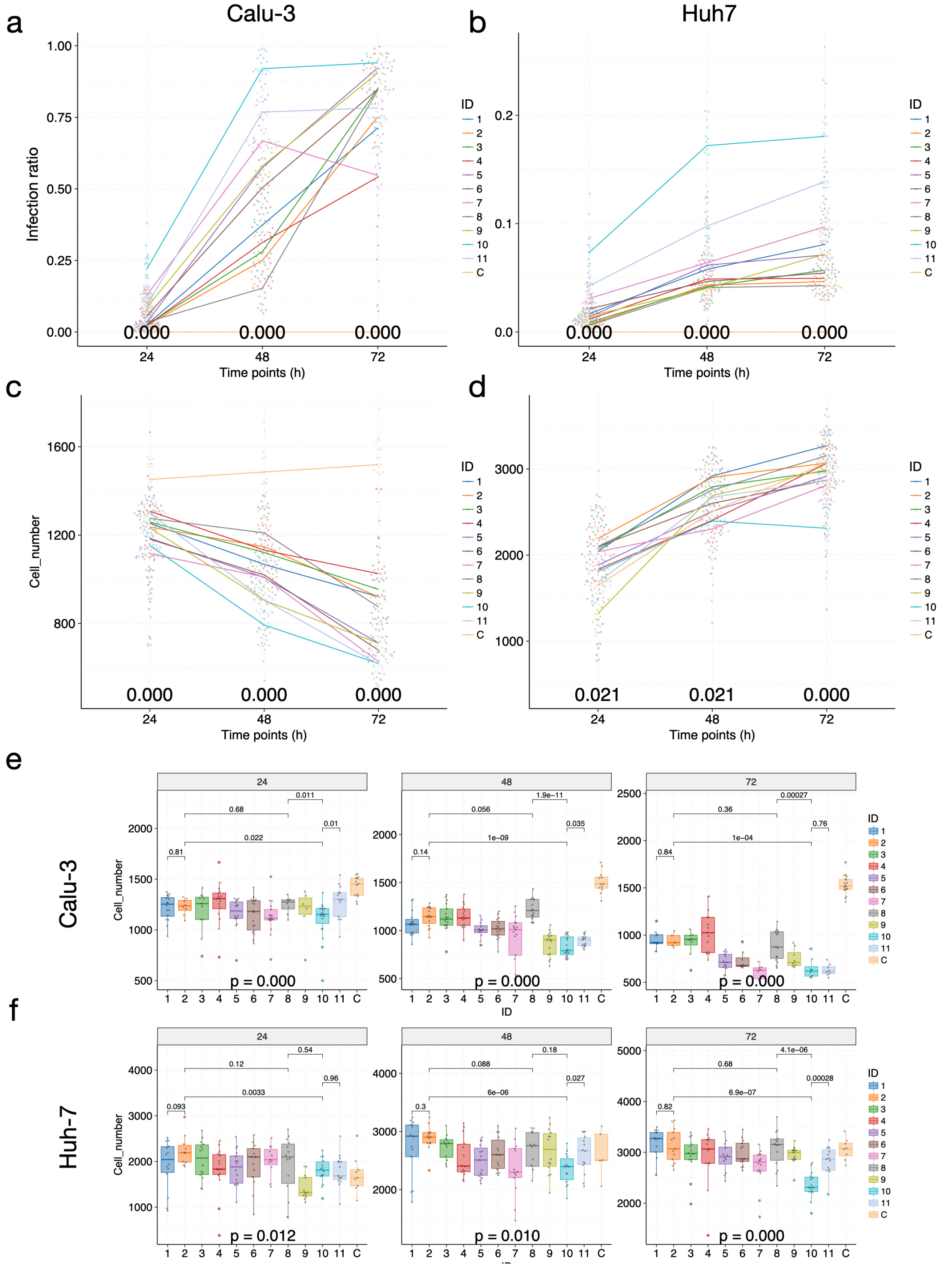

a
Calu-3
Infection ratio
Time points (h)
0.000
0.000
0.000
b
Huh7
Time points (h)
0.000
0.000
0.000
c
Cell_number
Time points (h)
0.000
0.000
0.000
d
Time points (h)
0.021
0.021
0.000
ID
e
Calu-3
Cell_number
24
48
72
p = 0.000
p = 0.000
p = 0.000
f
Huh-7
Cell_number
24
48
72
p = 0.012
p = 0.010
p = 0.000

Supplementary figure S6. Infection ratios and total cell numbers of Calu-3 and Huh-7 cells when infected by the 11 viral isolates. (a-b) The time-series plot of infection ratios in Calu-3 (a) and Huh-7 (b) cells when infected by the 11 viral isolates. (c-f) The time-series plot of total cell numbers (counted per field) in Calu-3 (c) and Huh-7 (d) cells when infected by the 11 viral isolates. The box plots of total cell numbers per image in Calu-3 (e) and Huh-7 (f) cells when infected by the 11 viral isolates. Note that the total cell number increases over time for controls in both cell lines. At each timepoint, a p-value was calculated using the ANOVA method to compare the means of C_t values of different viral isolates. Pair-wise p-values were calculated using the t-test and adjusted p-values are shown.

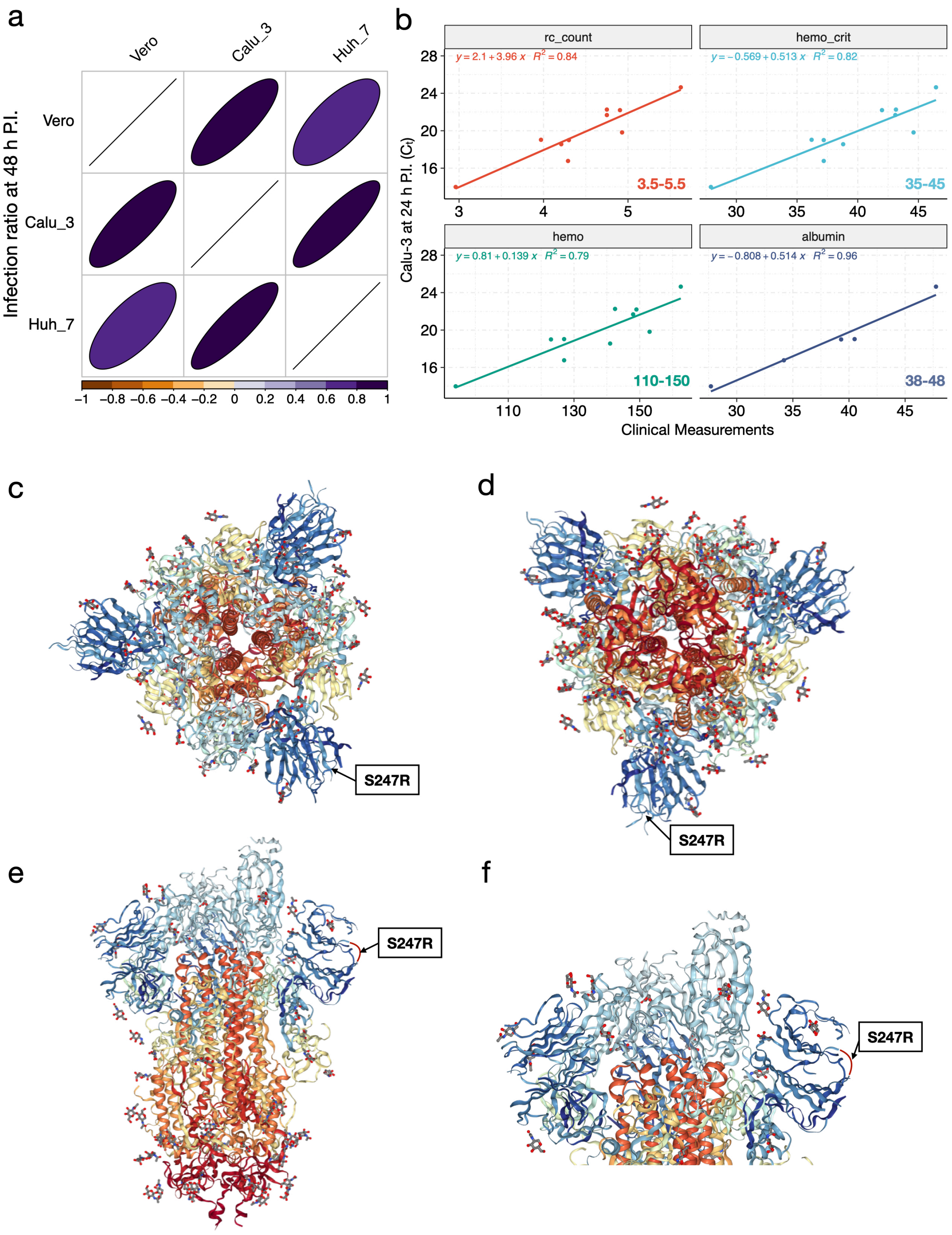
a
Vero
Calu_3
Huh_7
Infection ratio at 48 h P.I.
−1 −0.8 −0.6 −0.4 −0.2 0 0.2 0.4 0.6 0.8 1
b
rc_count
y = 2.1 + 3.96 x R^2 = 0.84
3.5-5.5
hemo_crit
y = −0.569 + 0.513 x R^2 = 0.82
35-45
hemo
y = 0.81 + 0.139 x R^2 = 0.79
110-150
albumin
y = −0.808 + 0.514 x R^2 = 0.96
38-48
Calu-3 at 24 h P.I. (Ct)
Clinical Measurements
c
S247R
d
S247R
e
S247R
f
S247R

Supplementary figure S7. Mutational consequences of patient-derived SARS-CoV-2 viral isolates. (a) Variation patterns in infection ratio of the 11 viral isolates were highly consistent in Vero, Calu-3, and Huh-7 cell lines. The correlation coefficients are color-coded according to the bottom legend and also visualized in ellipses, with the circularity inversely related to the correlation coefficient; only correlation coefficients with adjusted p-values < 0.05 were shown. (b) Variations in viral titers correlated with the variations in patients' clinical data. Regression functions and the normal ranges of each clinical variable (for women) are shown on each panel; units omitted for consistency. (c-f) The top (c), bottom (d), side (e), and close-up view (f) were provided. Note that the actual position of S247 was not determined in the original structure, hence a small red arc was in place to represent the flexible loop region in (c) and (d). The protein complex is trimeric, but only one of the three mutations were labeled. The 3D structure of the S protein was visualized and downloaded from https://www.rcsb.org/3d-view/6VSB/1.

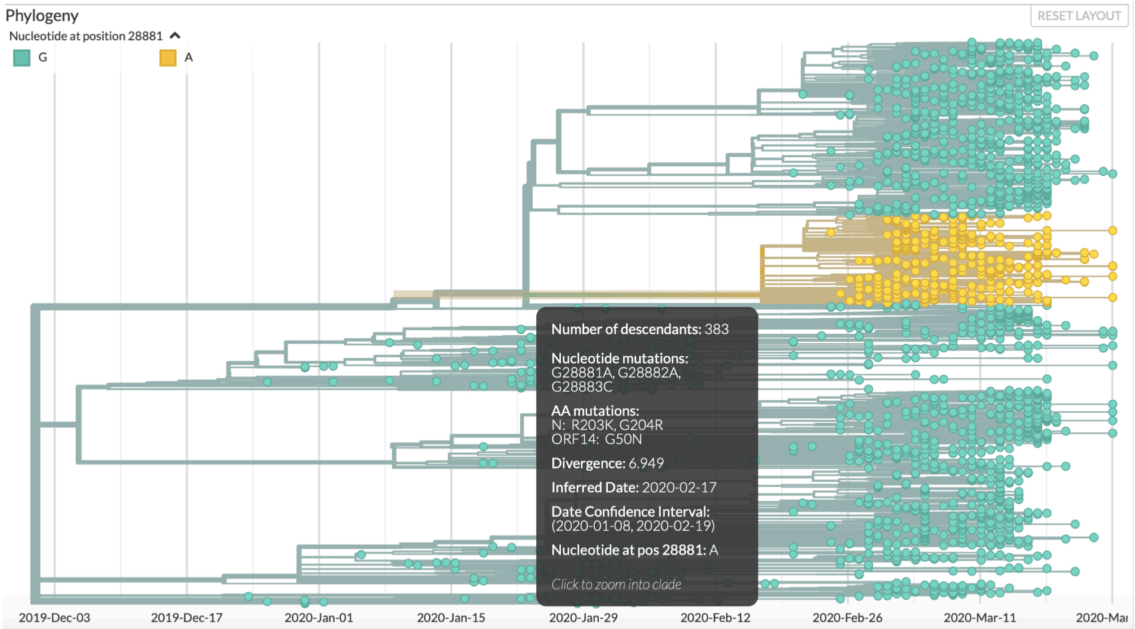

A subclade of the S-D614G group

Analyses downloaded on 3/31/2020

Supplementary figure S8. The trinucleotide mutation (G28881A, G28882A, and G28883C) was identified in the GISAID dataset and is shared by a large cluster of viral isolates within the S-D614G group (European clade), related to Figure 1.
